# Supplementary material for: The Therapeutic Effects of Blueberry-Treated Stem Cell-Derived Extracellular Vesicles in Ischemic Stroke
Source: Int J Mol Sci. 2024 Jun 8;25(12):6362. doi: 10.3390/ijms25126362 (PMC11203670; doi:10.3390/ijms25126362)
Supplement: Supplementary file 1 [file ijms-25-06362-s001.zip › ijms-2989047-supplementary.pdf]

Supplementary Materials for

The Therapeutic Effects of Blueberry-Treated Stem Cell-Derived Extracellular Vesicles in Ischemic Stroke

Eunjae Jang[1,2], Hee Yu[1,2], Eungpil Kim[3], Jinsu Hwang[1], Jin Yoo[4], Jiyun Choi[1], Han-Seong Jeong[1,#], Sujeong Jang[1,*]

Materials and Methods

S.1. Quantitative PCR (qPCR)

To investigate the expression of target genes, we performed qPCR. Following the manufacturer's instructions, total RNA was isolated using TRIzol reagent (Invitrogen; Thermo Fisher Scientific, Inc.) from HT-22 cells treated with A-EV and B-EV after recovery from OGD conditions. According to the manufacturer's instructions, cDNA was synthesized by adding recombinant RNase inhibitor (TaKaRa Bio Co.), GoScript oligo(dT) (Promega Co., Madison, USA), and GoScript Enzyme Mix (Promega Co.). qPCR analyses were performed using a LightCycler 480 II (Roche Holding AG., Basel, Swiss) and SYBR Green Premix Ex Taq (Takara Bio Co.) at the suggested annealing temperature of 60 ℃. Three replicate reactions were analyzed to each sample. All primers were purchased from Bioneer and CosmoGenetech (Seoul, Korea) and listed in **Table S1**.

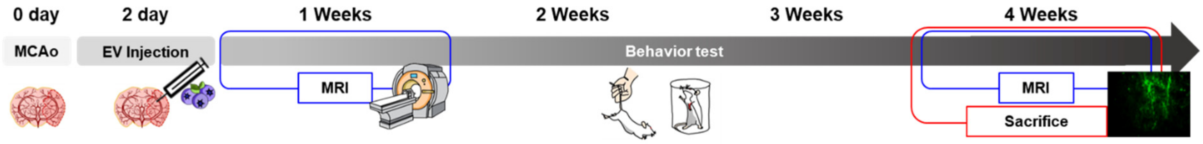

Figure. S1. The experimental procedures for the MCAo animals. All experiments were performed following this schedule. B-EVs were injected two days after the MCAo. We performed the cylinder test every week, and performed the MRI the first and fourth week after surgery. The tissues from animals were collected for a TUNEL assay and immunohistochemistry four weeks after surgery.

Table S1. Sequence of qPCR primers.

Gene	Forward (5'-3')	Reverse (5'-3')	PMID (Ref)
SUMO2	GGCAGCCAATCAACGAAACA	TCAACCAGAACAGAACATCGGT	NM_133354.2[98]
FN1	ATGAGAAGCCTGGATCCCCT	GAGAGCTTCCTGTCCTGTCT	NM_001276413.1[107]
THBS1	AGCACTCGGCCTTTAACGAA	GCCTCGAAGAAGTTCTGGCT	NM_001313914.1[101]
CYTB	ACTGTTCGCAGTCATAGCCA	GCGAAGAATCGGGTCAAGGT	KX790793.1[102]
NEAT1	GCCTCAGACCCTCAGTTTCC	ATGCCATCCAGGTTCCAAGG	NR_131212.1[103]
PRDX1	CCAAGTGATTGGCGCTTCTG	GGTGCGCTTGGGATCTGATA	NM_011034.5[106]

Abbreviations: *SUMO2*, Small ubiquitin like modifier 2; *FN1*, Fibronectin1; *THBS1*, Thrombospondin-1; *CYTB*, Cytochrome b; *NEAT1*, Nuclear paraspeckle assembly transcript 1; *PRDX1*, Peroxiredoxin 1

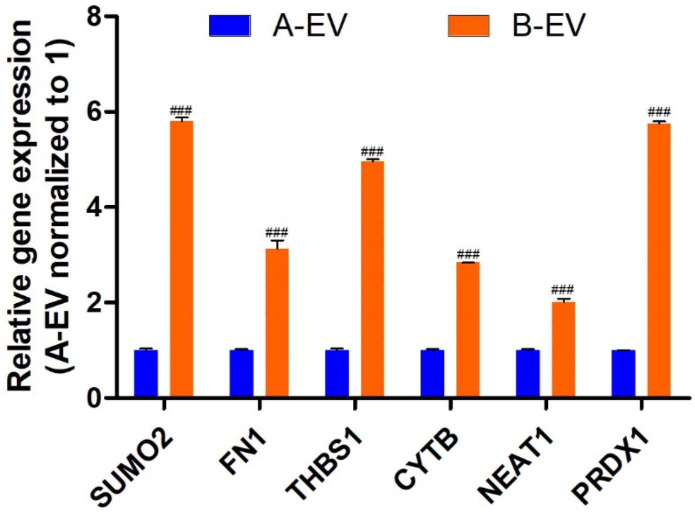

Figure. S2. The gene expression of A-EV and B-EV was confirmed through PCR following OGD. The expression levels of various genes were assessed in different experimental groups. The qPCR data was normalized to the A-EV as 1. In the B-EV group, the expression levels were as follows: *SUMO2*, 5.8 ± 0.04; *FN1*, 3.13 ± 0.1; *THBS1*, 4.95 ± 0.02; *CYTB*, 2.84 ± 0.01; *NEAT1*, 2.01 ± 0.04; and *PRDX1*, 5.74 ± 0.27. Statistical significance compared to the A-EV group was observed ($^{###}p < 0.001$).

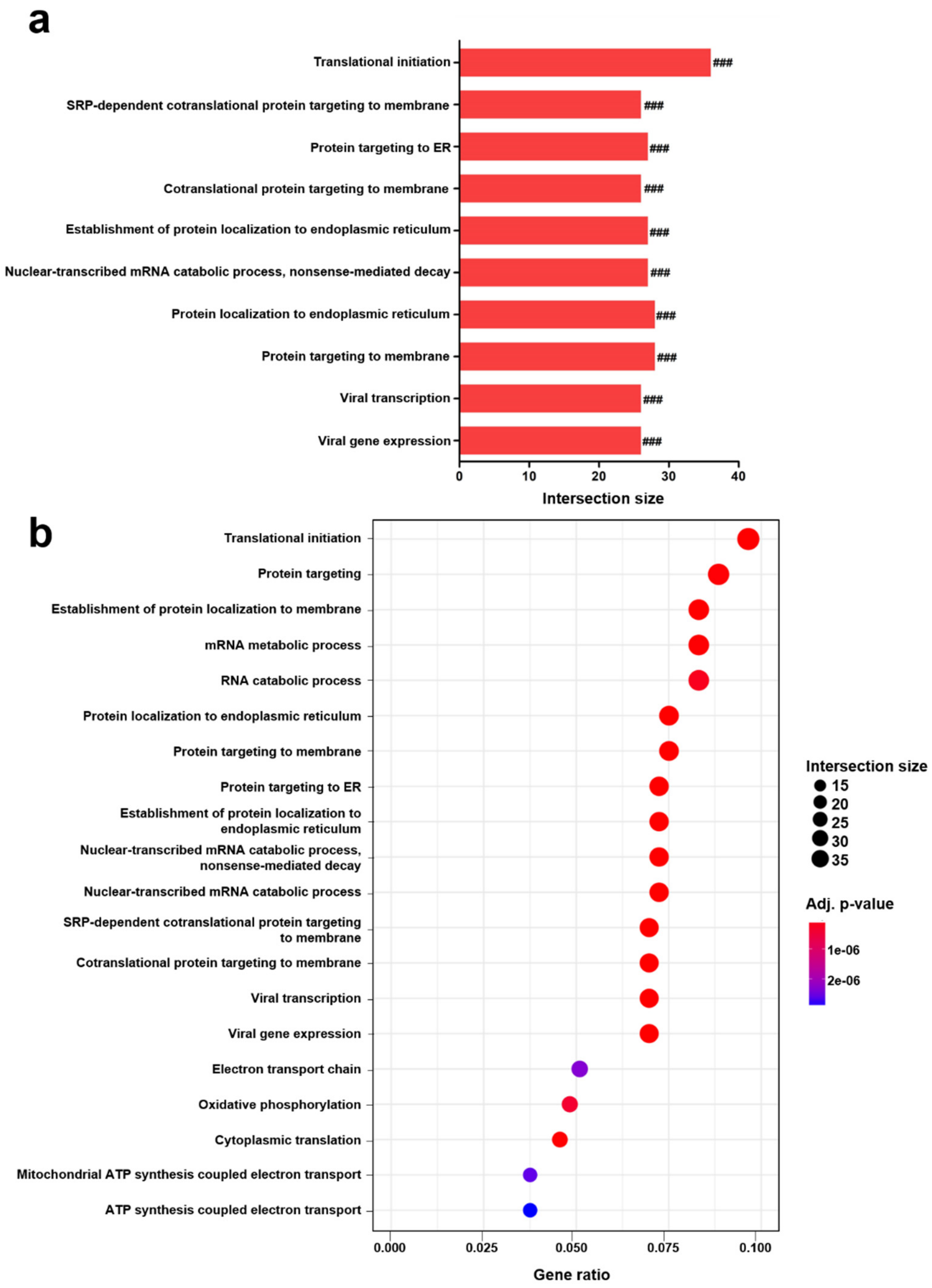

Figure. S3. Gene ontology analysis of B-EVs. The top 10 up-regulated terms in functional groups, a) biology process and b) all the up-regulation term in functional groups ($^{###}p < 0.001$ compared to the A-EVs).

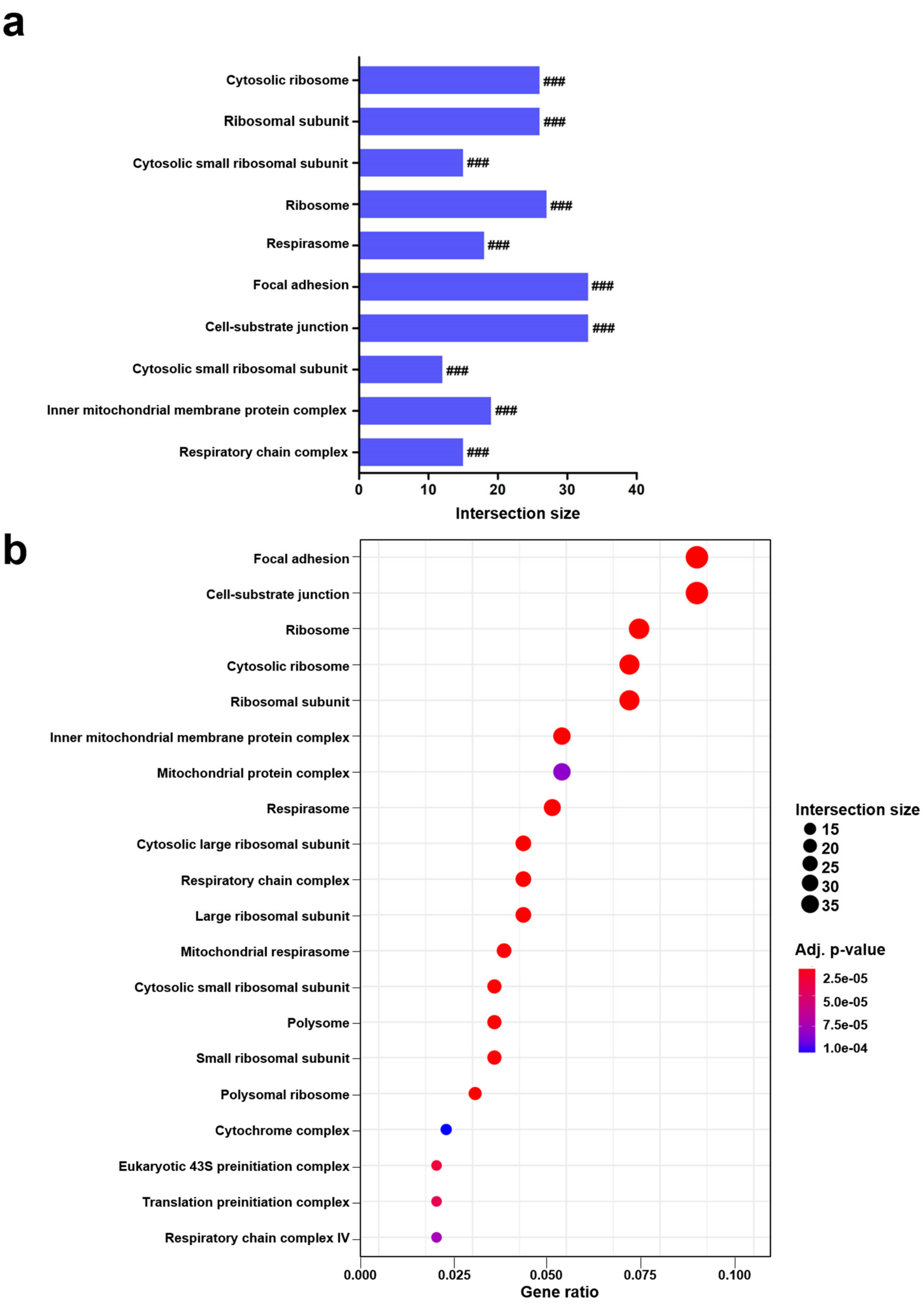

Figure. S4. Gene ontology analysis of B-EVs. The top 10 up-regulated terms in functional groups, a) Molecular function, b) all the up-regulation term in functional groups ($^{###}p < 0.001$ compared to the A-EVs).

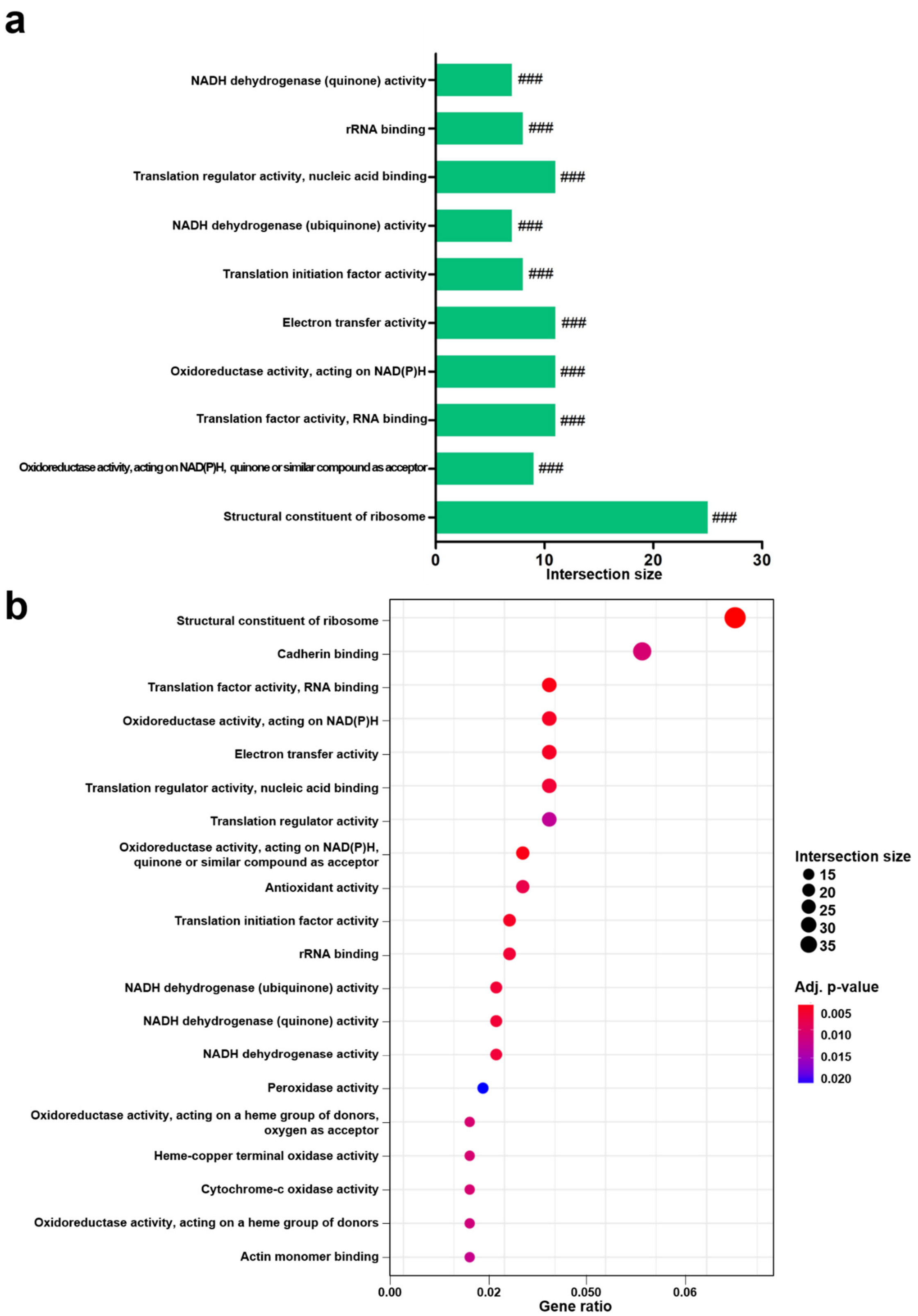

Figure. S5. Gene ontology analysis of B-EVs. The top 10 up-regulated terms in molecular function groups, a) cellular component, b) all the up-regulation term in functional groups ($^{###}p < 0.001$ compared to the A-EVs).
